# Replication stress, DNA damage signalling, and cytomegalovirus infection in human medulloblastomas

**DOI:** 10.1002/1878-0261.12061

**Published:** 2017-06-17

**Authors:** Jiri Bartek, Olesja Fornara, Joanna Maria Merchut‐Maya, Apolinar Maya‐Mendoza, Afshar Rahbar, Giuseppe Stragliotto, Helle Broholm, Mikael Svensson, Astrid Sehested, Cecilia Söderberg Naucler, Jiri Bartek, Jirina Bartkova

**Affiliations:** ^1^ Department of Medicine Unit of Microbial Pathogenesis Karolinska Institutet Stockholm Sweden; ^2^ Danish Cancer Society Research Center Copenhagen Denmark; ^3^ Department of Neurosurgery Karolinska University Hospital Stockholm Sweden; ^4^ Department of Neurosurgery Copenhagen University Hospital Rigshospitalet Denmark; ^5^ Department of Pathology Copenhagen University Hospital Rigshospitalet Denmark; ^6^ Department of Paediatrics and Adolescent Medicine Copenhagen University Hospital Rigshospitalet Denmark; ^7^ Division of Genome Biology Department of Medical Biochemistry and Biophysics Science for Life Laboratory Karolinska Institute Stockholm Sweden

**Keywords:** 53BP1, ATM, ATR and p53 tumour suppressors, brain tumours, cytomegalovirus proteins, endogenous DNA damage signalling, oxidative stress, replication stress

## Abstract

Medulloblastomas are the most common, and often fatal, paediatric brain tumours that feature high genomic instability, frequent infection by human cytomegalovirus (HCMV) and resistance to radiation and chemotherapy. The causes of the pronounced chromosomal instability and its potential links with HCMV infection and/or resistance to genotoxic therapies remain largely unknown. To address these issues, here we have combined immunohistochemical analysis of a series of 25 paediatric medulloblastomas, complemented by medulloblastoma cell culture models including experimental HCMV infection. Using eight established immunohistochemical markers to assess the status of the DDR machinery, we found pronounced endogenous DNA damage signalling (γH2AX marker) and robust constitutive activation of both the ATM‐Chk2 and ATR‐Chk1 DNA damage checkpoint kinase cascades, yet unexpectedly modest p53 tumour suppressor activation, across our medulloblastoma cohort. Most tumours showed high proliferation (Ki67 marker), variable oxidative DNA damage (8‐oxoguanine lesions) and formation of 53BP1 nuclear ‘bodies’, the latter indicating (along with ATR‐Chk1 signalling) endogenous replication stress. The bulk of the clinical specimens also showed expression of HCMV immediate early and late proteins, in comparative analyses using three immunohistochemical protocols. Cell culture experiments validated the chronic endogenous replication stress in medulloblastoma cell lines and showed sharply differential, intriguing responses of normal cells and medulloblastoma cells to HCMV infection, including differential subcellular mislocalization and enhancement of replication stress‐related 53BP1 body formation, the latter in cell‐non‐autonomous manner. Overall, our results strongly indicate that in human medulloblastomas, the DDR checkpoint barrier is widely activated, at least in part due to replication stress. Furthermore, we propose that unorthodox p53 defects other than mutations may allow high proliferation despite the ongoing checkpoint signalling and that the highly prevalent HCMV may impact the medulloblastoma host cell replication stress and DNA repair. Collectively, the scenario we report here likely fuels genomic instability and evolution of medulloblastoma resistance to standard‐of‐care genotoxic treatments.

**Abbreviations**
53BP1: p53‐binding protein 1
ATCC: American Type Culture Collection
ATM: ataxia telangiectasia mutated
ATR: ataxia telangiectasia mutated and Rad3 related
Chk1: checkpoint kinase 1
Chk2: checkpoint kinase 2
COX‐2: cyclooxygenase‐2
DDR: DNA damage response
FBS: fetal bovine serum
FISH: fluorescence in situ hybridization
GFAP: glial fibrillary acidic protein
HCMV: human cytomegalovirus
IDH: isocitrate dehydrogenase
IE72: immediate early 72
Ini‐1: integrase interactor 1
MAP‐2: microtubule‐associated protein 2
MDC1: mediator of DNA damage checkpoint protein 1
MOI: multiplicity of infection
NGS: next‐generation sequencing
Olig2: oligodendrocyte transcription factor 2
PBS: phosphate‐buffered saline
PGE2: prostaglandin E2
SHH: sonic hedgehog
WNT: wingless
γH2AX: phosphorylated histone H2AX

## 1 Introduction

Human paediatric brain malignancies encompass a diverse group of tumours originating from various cell types, featuring a spectrum of genetic and epigenetic alterations and pathogenic trajectories. Given the great variation in their key pathobiological properties, highly variable responses to standard‐of‐care treatment modalities and often grave prognosis, brain tumours pose a number of challenges in the clinic as well as in terms of understanding their molecular pathogenesis (Downing *et al*., 2012; Pui *et al*., 2011). In this study, we focus on paediatric medulloblastomas, in an attempt to address two of the outstanding puzzles in the field of brain cancer research: (a) the occurrence and source of endogenous genotoxic stress that results in DNA damage checkpoint activation as a potential biological barrier against progression of primary tumours (Bartek *et al*., 2007) and (b) the evidence for involvement of human cytomegalovirus (HCMV) in pathogenesis of these malignancies (Baryawno *et al*., 2011; Cobbs, 2013). Furthermore, we consider a possible link between these two phenomena. Our motivation to carry out this investigation reflected the need to provide novel pathobiological insights into human medulloblastomas, but also the fact that the extent of endogenous genotoxic stress and cellular responses to such DNA damage, along with the presence and potential role of HCMV in tumours, are highly relevant for treatment strategies in contemporary oncology (Bartek *et al*., 2012; Cobbs, 2013; Lecona and Fernandez‐Capetillo, 2014; Lord and Ashworth, 2012; Northcott *et al*., 2012; Soderberg‐Naucler and Johnsen, 2015; Taylor *et al*., 2012).

Medulloblastomas are the second most frequent malignant brain tumours in children. Chemotherapy and radiotherapy are used as standard‐of‐care treatment modalities, yet a large fraction of medulloblastomas show resistance to such genotoxic therapies. Given the challenge that medulloblastoma chemo‐radioresistance poses to paediatric oncologists, and the evidence for genetic instability among these brain malignancies (Northcott *et al*., 2012; Polkinghorn and Tarbell, 2007), we argued that potential endogenous activation and/or malfunctioning of the cellular DNA damage‐response (DDR) machinery (Jackson and Bartek, 2009; Kastan and Bartek, 2004) might play a role in molecular pathogenesis of medulloblastomas and also impact the therapeutic responses of individual patients. The fact that there has been no report so far on the status of key DNA damage‐response pathways in human medulloblastomas, in contrast to such studies being available for gliomas and paediatric intracranial germ‐cell tumours (Bartkova *et al*., 2010, 2014), provided further impetus to carry out this study. Apart from its relevance for the outcome of chemotherapy and radiation treatments, the DNA damage checkpoint activation unrelated to therapy, indeed commonly preceding any genotoxic treatments, has been reported for a broad range of human tumour types, reflecting cancer cells' responses to their endogenous DNA damage caused largely by replication and oxidative stresses triggered by activated oncogenes or loss of some tumour suppressors (Bartkova *et al*., 2005b, 2006; Di Micco *et al*., 2006; Gorgoulis *et al*., 2005; Lecona and Fernandez‐Capetillo, 2014). Such DNA damage checkpoint activation involves two major DDR signalling cascades of ATM‐Chk2 and ATR‐Chk1 kinases, and it appears to serve as an intrinsic biological barrier against activated oncogenes and tumour progression, triggering senescence or death of the nascent cancer cells through p53‐dependent and p53‐independent pathways downstream of ATM and ATR signalling (Bartek *et al*., 2007; Halazonetis *et al*., 2008). At the same time, such cellular environment of ongoing chronic DNA damage signalling provides a selection pressure that favours outgrowth of tumour cells capable of bypassing this DDR barrier, such as cancer cells with mutant p53, ATM, Chk2, or aberrant DNA repair pathways, among other adaptive alterations. While this DDR barrier concept appears to be applicable to all types of major human epithelial malignancies (Bartek *et al*., 2007; Halazonetis *et al*., 2008), its general applicability to brain tumours remains uncertain. Indeed, while widespread endogenous DDR activation has been reported for human gliomas (Bartkova *et al*., 2010; Rasmussen *et al*., 2016), it was largely absent among paediatric intracranial germ‐cell tumours (Bartkova *et al*., 2014), implying that cell of origin and oncogenic events, rather than the brain ‘environment’ *per se* dictate whether or not intracranial tumours, trigger the intrinsic DDR barrier. To what extent is the DNA damage checkpoint machinery activated in human medulloblastomas and whether their pathogenesis involves endogenous replication or oxidative stresses are currently unknown. To address these fundamental issues was the first aim of this study.

The second major aim of this investigation was to reassess the topical yet currently partly controversial issue of cancer‐associated presence of human cytomegalovirus (HCMV), through analysis of our cohort of paediatric medulloblastomas, complemented by medulloblastoma cell lines as a model system amenable for experimentation, including direct viral infection. HCMV has been detected in clinical specimens of both human glioblastomas and medulloblastomas at the level of HCMV nucleic acids as well as immediate early and late HCMV proteins, respectively (Baryawno *et al*., 2011; Soderberg‐Naucler and Johnsen, 2012, 2015). In addition, analogous to several other types of human cancer (Harkins *et al*., 2002; Michaelis *et al*., 2009; Samanta *et al*., 2003; Taher *et al*., 2014), HCMV has been reported to exert an oncomodulatory role in brain tumours, including promotion of cancer ‘stemness’ features, telomerase activation, immune suppression and enhancement of proliferation‐stimulating factors such as COX‐2 or PGE2 (Fornara *et al*., 2016; Straat *et al*., 2009; Zhu *et al*., 2002). Notably, a notion of HCMV as a candidate therapeutic target in brain tumours has been highlighted by recent studies, in which treatment with the antiviral drug ganciclovir showed promising antitumorigenic effects in both glioblastoma and medulloblastoma cell lines and xenografts, and in clinical settings, ganciclovir treatment correlated with prolonged survival of human patients with glioblastoma (Cobbs, 2014; Soderberg‐Naucler *et al*., 2013). In contrast to such promising results provided by the bulk of HCMV studies performed so far, reports from several laboratories expressed scepticism about HCMV protein detection in human cancer (Baumgarten *et al*., 2014; Sardi *et al*., 2015; Yamashita *et al*., 2014). Faced with such apparent discrepancy with regard to the prevalence of HCMV in human tumours, we decided to employ and compare several complementary immunohistochemical approaches to assess their respective suitability for detecting HCMV early and late proteins in our series of formalin‐fixed, paraffin‐embedded archival specimens of human medulloblastomas.

Last but not least, our data obtained by pursuing the above two aims allowed us to search for any potential correlations between the extent of DDR activation and HCMV prevalence, as well as any relationships of either DDR status or HCMV with the clinical parameters and molecular subtypes of the medulloblastomas in our cohort. These additional questions are therefore also addressed in this study. Related to the molecular subtyping of medulloblastomas, recent efforts have identified key mutations in the Hedgehog and Wnt developmental pathways, as well as in additional oncogenes, and these currently form the basis for a subclassification scheme that is, however, still in progress and requires further research (Taylor *et al*., 2012). Neither the status of DDR machinery activation, nor any potential relationships between the DDR status and patients' clinical course, molecular subtypes or HCMV status has been so far elucidated in medulloblastomas. Our results from these analyses, complemented by cell culture model experiments with human medulloblastoma cell lines, are presented below and their significance discussed within the context of the current understanding of brain tumour pathogenesis and relevance for therapy.

## 2 Materials and methods

### 2.1 Patient population

This study includes a cohort of paediatric medulloblastoma patients treated at the Neurosurgical and Paediatric Oncology Department of the Copenhagen University Hospital (Rigshospitalet). Between 1998 and 2009, 25 consecutive paediatric patients (<16 years) were treated for a newly diagnosed medulloblastoma, 24 of whom were patients undergoing first‐time surgical resections, while one (patient no. 16) was treated with secondary surgery due to tumour recurrence, with the first surgery performed 1 year previously at a foreign institution. At a later stage, additional three patients in the cohort were treated with secondary surgery due to tumour recurrence (patients no. 1, 23, 24). Paraffin‐embedded archival tissue was obtained from all the above, except from the primary surgery of patient no. 16 performed abroad. There were no adult (>16 years) cases of medulloblastoma treated during this time period. Clinicopathological data were collected from the hospital patient charts (see Table 1 and Results Section 3.1). Patient characteristics and preoperative status, including potential radiological signs of metastatic disease, were registered. The patients were monitored through medical chart reviews from the Department of Neurosurgery and Paediatric Oncology.

**Table 1:** Demographic data about the cohort of paediatric medulloblastoma patients (*n* = 25)

Patient no.	Age at surgery	Sex	Risk group	Outcome	Survival	Metastasis (Yes/No)
1	10 years	M	SR	DOD	46	N
2	4 years	M	SR	NED	191+	N
3	5 months	F	HR	DOD	2	N
4	11 years	F	SR	NED	121+	N
5	6 years	M	SR	NED	138+	N
6	14 years	M	SR	NED	139+	N
7	7 years	M	SR	NED	160+	N
8	4 years	F	SR	NED	164+	N
9	9 years	M	HR	NED	129+	Y
10	11 years	M	SR	NED	126+	N
11	2 years	F	HR	DOD	7	Y
12	8 years	M	HR	DOD	1	N
13	11 years	M	HR	DOD	1	Y
14	3 years	M	SR	NED	130+	N
15	4 years	M	SR	NED	110+	N
16	9 years	M	SR	NED	106+	N
17	9 years	M	SR	NED	106+	N
18	3 years	M	SR	DOD	33	N
19	14 years	F	HR	NED	80+	Y
20	2 years	M	HR	DOD	1	N
21	15 years	F	HR	DOD	58	Y
22	4 years	F	HR	DOD	62	Y
23	5 years	M	HR	DOD	48	Y
24	5 years	F	HR	DOD	16	Y
25	4 years	F	SR	NED	62+	N

HR, high risk; SR, standard risk; NED, no evidence of disease; DOD, dead of disease.

### 2.2 Pathology and immunohistochemistry

Medulloblastomas are embryonal neuroepithelial tumours composed of small blue cells which after histomorphological criteria are classified in four defined subgroups as either classic, desmoplastic/nodular, medulloblastoma with extensive nodularity or large cell/anaplastic medulloblastoma, according to the WHO 2016 classification (Louis *et al*., 2016). Furthermore, medulloblastomas harbour characteristic genetic changes in distinct molecular pathways and are genetically classified into four major groups: (1) WNT‐activated, (2) SHH‐activated and the more complex non‐WNT/non‐SHH‐activated groups 3 and 4 (Taylor *et al*., 2012). This subclassification has shown clinical utility, and in the latest WHO edition (2016), the diagnosis of medulloblastoma is an integrated strategy that incorporates both the histopathological subtype and the genetic subtype (Louis *et al*., 2014).

The diagnosis of a medulloblastoma demands a panel of immunohistochemical staining assays, FISH analysis and molecular investigation (mostly GFAP, p53, MAP‐2, synaptophysin, Ini‐1, Olig2, IDH, ATRX, CD56, Lin28, CK, FISH for MYC amplification, methylation assay or NGS) to subclassify both histologically and genetically and exclude other paediatric and especially other embryonal tumours. In addition to these standardized automated immunohistochemical methods routinely used at Rigshospitalet in Copenhagen and performed on specimens from our cohort, molecular subtyping by investigation of expression of beta‐catenin and Yap1 has been performed, albeit the latter two assays on only 60% of the specimens (see Section 3).

To examine the expression of a wide range of DNA damage‐response proteins, their modifications (detection of phosphospecific epitopes) and DNA lesions due to oxidative damage (8‐oxoguanine), the full list of which is presented in Table 2, we employed our well‐established sensitive immunohistochemical staining protocol (Bartkova *et al*., 2005a, 2014). The latter method, optimized in the Bartek laboratory in Copenhagen, involves standard deparaffinization of the archival formalin‐fixed, paraffin‐embedded tissue sections, followed by antigen unmasking in the citrate buffer (pH6; NB: this step was omitted for 8‐oxoG and p53 staining), then overnight incubation with the primary mouse or rabbit antibody against the selected human protein or protein modification, and subsequent processing by the indirect streptavidin–biotin–peroxidase method using the Vectastain Elite kit (Vector Laboratories, Burlingame, CA, USA) and nickel sulfate‐based chromogen enhancement detection as previously described, without nuclear counterstaining (Bartkova *et al*., 2005a, 2014). For the detection of the HCMV immediate early and late proteins, we employed three immunohistochemical protocols, including the one above that was used also for the detection of the DDR proteins (referred to here as ‘Protocol A’). In ‘Protocol B’, the antigen unmasking step involved incubation of deparaffinized sections in the Tris/EDTA buffer (pH 9), while the rest of this Protocol was identical with Protocol A. Protocol C was based on the original method from the Cobbs laboratory for sensitive detection of the HCMV proteins (Cobbs *et al*., 2002, 2014), optimized for HCMV studies in human brain tumours in the Söderberg‐Naucler laboratory and involved robust enzymatic antigen unmasking steps and immunoperoxidase‐based detection with nuclear counterstaining (Baryawno *et al*., 2011; Rahbar *et al*., 2012, 2013). For negative controls, sections were incubated with nonimmune mouse or rabbit sera, while sections from human glioblastomas previously shown to express the studied proteins served as positive controls. The results were evaluated by two experienced researchers, including a senior oncopathologist, and the data were expressed in scoring categories based on the percentage of positive tumour cells within each lesion (see Section 3 for more details).

**Table 2:** Summary of immunohistochemical analyses of DNA damage‐response markers, proliferation and molecular subtypes among the medulloblastoma cohort

Patient no.	γH2AX	P‐CHK2	P‐ATM	P‐CHK1	P‐ATR	53BP1 (%)	CHK2 tot. (%)	ATM tot. (%)	Ki‐67 (%)	8‐oxo (%)	p53 (%)	Class/Group
1	C	B	C	C	C	80	75	70	20	9	1	3/4
2	C	B	B	C	C	80	70	70	15	15	5	n/a
3	D	B	B	D	D	80	75	70	56	12	4	Shh
4	C	C	C	C	C	80	75	70	15	6	7	3–4
5	C	C	C	C	C	80	75	80	14	15	10	n/a
6	C	B	C	C	C	80	70	70	35	9	4	3/4
7	C	B	C	C	C	80	70	70	20	6	1	3/4
8	C	C	C	C	C	75	80	75	30	15	5	3/4
9	C	C	C	C	C	80	80	80	25	40	1	n/a
10	D	C	C	D	D	80	72	70	25	15	30	Wnt
11	D	C	D	C	D	75	75	72	40	15	0.5	Shh
12	D	C	C	D	C	80	71	72	38	10	5	3/4
13	B	B	B	B	B	80	85	85	15	7	3	n/a
14	D	C	D	D	D	85	80	80	40	60	6	n/a
15	C	C	C	D	D	80	80	80	25	3	3	n/a
16	D	C	D	D	D	80	90	75	60	15	2	n/a
17	C	A	B	C	C	80	95	95	35	15	6	n/a
18	D	C	D	C	C	95	80	85	22	1	0.5	n/a
19	C	C	C	C	C	75	87	85	18	4	1	3/4
20	B	B	B	B	B	75	77	75	15	15	1	3/4
21	C	C	C	C	C	76	76	75	18	5	1	3/4
22	D	C	C	D	C	75	70	70	40	3	40	3/4
23	D	C	C	C	C	85	77	86	25	16	0.5	3/4
24	D	C	D	D	D	85	90	90	40	40	40	n/a
25	B	B	C	B	B	77	72	72	20	5	2	3/4

N/A, not available; Shh, sonic hedgehog; Wnt, wingless.

Classification: γH2AX, P‐CHK2, P‐ATM, P‐CHK1 and P‐ATR subdivided into categories according to fractions of positive tumour cells: 0–1% (A), 2–10% (B), 11–50% (C) and 51–100% (D).

### 2.3 Primary antibodies

Antibodies used in this study for immunohistochemical analysis included the following reagents: mouse monoclonal antibody to human phospho‐histone H2A.X (Ser 139) (Millipore, Temecula, CA, USA, clone JBW 301, diluted 1 : 2500), rabbit polyclonal antibody to human phosphorylated Chk2 (Thr 68) (Cell Signaling, Danvers, MA, USA, 1 : 1000), mouse monoclonal antibody to phosphorylated ATM (pS 1981) clone 7C10D8, gift from M. Kastan, St. Jude Children's Hospital, Memphis, TN, USA, 1 : 75), rabbit antiserum against phosphorylated CHK1 (Ser 317) (Cell Signaling, 1 : 2000), rabbit polyclonal antibody to phosphorylated ATR (Ser 428) (Cell Signaling, 1 : 500), mouse monoclonal antibody to 53BP1 (a gift from T. Halazonetis, 1 : 50), mouse monoclonal antibody to MDC1 (clone DCS 380, our own antibody, 1 : 250), mouse monoclonal antibody to Chk2 (clone DCS 270, our own antibody, 1 : 2000), rabbit monoclonal antibody to ATM (Abcam, Cambridge, UK clone Y170, 1 : 500), rabbit polyclonal antibody to Ki67 antigen (Leica, Wetzlar, Germany, 1 : 6000), mouse monoclonal antibody to 8‐oxoguanine (Rockland, Limerick, PA, USA, 1 : 1000), mouse monoclonal antibody to p53 (clone DO1, our own antibody, 1 : 3000), mouse monoclonal antibody to HCMV immediate early protein (Millipore‐Chemicon, Temecula, CA, USA, clone MAB 810R, 1 : 2000), mouse monoclonal antibody to HCMV late‐antigen protein (Millipore‐Chemicon, clone MAB 8127, 1 : 1000), mouse monoclonal antibody to beta‐catenin (BD, Santa Monica, CA, USA, clone 14, 1 : 100) and mouse monoclonal antibody to YAP1 (Abcam, clone GR245919, 1 : 200).

### 2.4 Cell culture and HCMV infection

Human medulloblastoma cell lines DAOY and D324 and human strain BJ of normal diploid primary fibroblasts (all from the ATCC) were grown in the RPMI 1640 medium with 10% fetal bovine serum and antibiotics. For the analysis of the cell cycle phase‐dependent 53BP1 nuclear bodies, exponentially growing cells on coverslips were fixed and examined by double immunofluorescence with antibodies to cyclin A (to identify cells in S/G2 phases) and 53BP1. In some experiments, the BJ fibroblasts and the DAOY medulloblastoma cells were infected with the human cytomegalovirus (strain VR1814) as described (Baryawno *et al*., 2011), and the expression of HCMV immediate early protein and 53BP1 was examined in a time course by double immunofluorescence of cells cultured and fixed on coverslips. All cell types were regularly tested to exclude mycoplasma infection. It is not necessary here; description of immunofluorescence in more detail is in point 2.5.

### 2.5 Immunofluorescence

The immunofluorescence technique analysing expression and subcellular localization of 53BP1, cyclin A or HCMV immediate early protein followed largely standard indirect immunofluorescence procedures described in our recent publications (Fornara *et al*., 2016; Lukas *et al*., 2011). For immunofluorescence analysis of HCMV‐infected cells, BJ normal fibroblasts and DAOY medulloblastoma cells grown on cover slips were infected with the HCMV VR1814 strain at MOI 3 in 2% FBS DMEM or RPMI medium. At indicated time points, cover slips were washed with PBS (Gibco, Waltham, MA, USA), fixed with 4% formaldehyde (VWR Chemicals) (15 min, room temperature), permeabilized with 0.5% Triton X‐100 (Merck, Darmstadt, Germany) in PBS (10 min, room temperature) and blocked in PBS containing 0.1% Triton X‐100 and 1% BSA (Sigma‐Aldrich, St. Louis, MO, USA) (15 min, room temperature). Cells were incubated with the 53BP1 (sc‐22760, Santa Cruz Biotech, Dallas, TX, USA) rabbit and the IE72 (MAB810R, Millipore) mouse primary antibodies for 1 h at room temperature. After washing with PBS, cells were stained with the goat anti‐rabbit AlexaFluor‐568 and goat anti‐mouse AlexaFluor‐488 secondary antibodies (Invitrogen, Hvidovre, Denmark) for 1 h at room temperature. Cell nuclei were visualized using Hoechst 33342 (Invitrogen) in PBS (5 min, room temperature). Cover slips were washed with PBS and ddH_2_O, air‐dried and mounted with ProLong Gold (Life Technologies, Danvers, MA, USA). Images were acquired using a LSM800 confocal microscope (Carl Zeiss, Oberkochen, Germany), a 63x/1.4 oil immersion objective (Carl Zeiss) and lsm zen software (Munich, Germany). Image analysis was performed using imagej software (Bethesda, MD, USA).

### 2.6 Data evaluation and microphotography

Evaluation of the immunohistochemical staining patterns on tissue sections was performed by at least two independent and very experienced observers, one of whom was a senior oncopathologist. The scoring reflected the percentage of positive cells, the intensity of staining and the overall patterns, and the scoring categories for the individual ‘markers’ are explained in footnotes to Table 2. More detailed assessment of the immunohistochemical results, including subcellular localization and heterogeneity of the individual staining patterns, is provided in the results section, and representative examples of images from these analyses are shown in the main figures. Both evaluation and photography were performed using microscopes and photography equipment from Zeiss.

Evaluation of the immunofluorescence data on 53BP1 in relation to HCMV infection of cultured cells is explained in the legend to Fig. 8.

### 2.7 Ethical approval

The Regional Ethics Committee in Copenhagen, Denmark, approved the study (Ref. H‐6‐2014‐010).

## 3 Results

### 3.1 Patient characteristics and the extent of DNA damage signalling (γH2AX marker) in medulloblastomas

In order to address the aims of this investigation as outlined in the Introduction, we first retrieved a panel of archival medulloblastoma tissue blocks for a series of 25 paediatric patients and clinical information about this cohort. Overall, 16 male and nine female patients were included in this study (see Table 1), with a median age of 6 years (range, 5 months ‐ 15 years) at the time of surgery. Of the 25 patients, nine had metastatic disease at various intracranial locations at the time of presentation: medulla (*n* = 6), cerebellum (*n* = 1), hypophyseal tract (*n* = 1) and the corpus pineale region (*n* = 1). One patient received neoadjuvant therapy in the form of radiation therapy. After surgery, from which the specimens available for our analyses were obtained, the patients were stratified into high risk (HR, *n* = 10) vs. standard risk (SR, *n* = 15) group, the latter being defined by the following strict criteria: total or subtotal tumour resection, no visible metastases on craniospinal magnetic resonance imaging (MRI) and no meningeal dissemination on postoperative lumbar puncture CSF cytology (Gnekow, 1995). Three patients deceased shortly after surgery due to sepsis and multiorgan failure in one case, and haematoma/swelling with subsequent incarceration in two cases. During the time span for inclusion, different treatment regimens have been employed, resulting in heterogeneous treatment characteristics in our material. Nevertheless, all patients received an adjuvant regimen comprising radiation and chemotherapy as standard, besides a few exceptions; one patient received preoperative chemotherapy (no. 1) and two (no. 16 and 24) received only chemotherapy as initial adjuvant treatment without radiation (see also Section 2 and Table 1).

**Figure 1:**
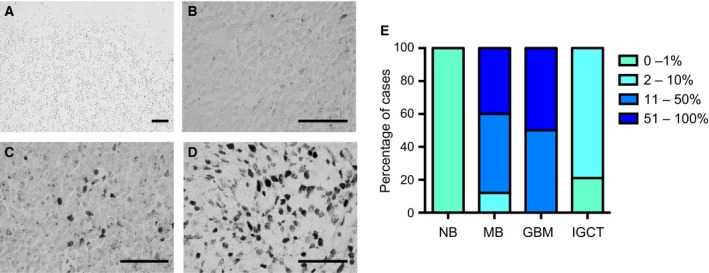
Immunohistochemistry of the γH2AX marker. Example of immunoperoxidase staining (negative γH2AX) in normal human cerebellum (A), and representative images documenting low (B), medium (C) and high (D) nuclear expression patterns of γH2AX among medulloblastomas (B–D). For A–D, the sensitive method without nuclear counterstaining (Protocol ‘A’ in the Section 2) was used, and the scale bars represent 50 µm. (E): Graphical summary of γH2AX staining patterns in normal human brain (NB,* n* = 15), glioblastoma multiforme (GBM,* n* = 60), intracranial germ‐cell tumours (IGCT,* n* = 19) and medulloblastoma (MB,* n* = 25, this study) assessed by semiquantitative immunoperoxidase analysis based on the proportion of positive cells, where the four categories of marker positivity (as indicated in E) reflect the percentage of positive cells within a lesion (the data for GBMs and paediatric IGCTs are from Bartkova *et al*., 2014).

Given the absence of information about endogenous DNA damage checkpoint activation in medulloblastomas, we first employed immunohistochemical examination of the extent of phosphorylated histone H2AX, the so‐called γH2AX marker (Bonner *et al*., 2008; Bartkova *et al*., 2005a,b) widely used to globally assess the activity of the apical DNA damage‐response kinases, including ATM and ATR that phosphorylate serine residue 139 of histone H2AX in the vicinity of DNA lesions. While no γH2AX staining was observed in the sections of normal human brain, including cerebellum from age‐matched subjects, a widely heterogeneous and often strong nuclear γH2AX staining was detected among medulloblastomas. Representative examples of the γH2AX staining patterns are shown in Fig. 1A–D. Furthermore, the overall results from a semiquantitative analysis of the immunohistochemical patterns, performed by an experienced oncopathologist, are presented in a graphical summary (Fig. 1E) and also listed for the individual patients in Table 2. Notably, the extent of the γH2AX marker positivity in our cohort of medulloblastomas was much greater than among human paediatric intracranial germ‐cell tumours, and only moderately lower compared with human glioblastomas, as examined by the same immunohistochemical protocol and evaluated by the same pathologist (Fig. 1E).

In summary, these results indicated that the endogenous DNA damage signalling documented by the γH2AX marker was robustly detectable in human paediatric medulloblastomas, thereby providing a rationale for further examination of the candidate checkpoint cascades, the status of key DDR factors and potential stresses behind such ‘spontaneous’ activation of the DDR machinery.

### 3.2 Widespread endogenous activation of the ATM‐Chk2 and ATR‐Chk1 signalling cascades in medulloblastomas

**Figure 2:**
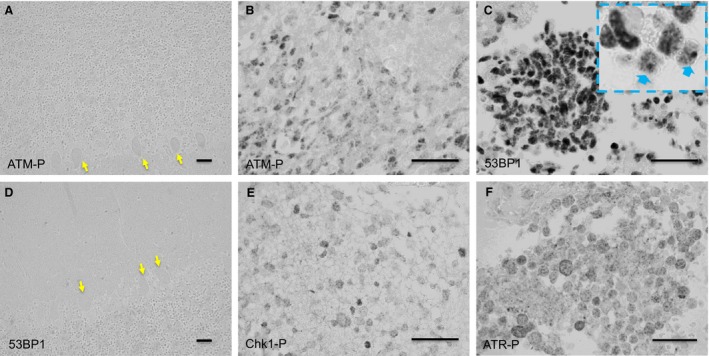
Immunohistochemical detection of various activated DDR kinases and 53BP1 by IHC staining Protocol ‘A’. Normal cerebellum is negative (A), while medulloblastoma heterogeneously positive (B) when stained for activated, phosphorylated ATM (ATM‐P); (C): medulloblastoma with strong nuclear positivity for 53BP‐1, and detectable nuclei with larger 53BP1 ‘bodies’ – see insert and blue arrows; normal cerebellum is negative (D), while medulloblastoma heterogeneously positive (E) when stained for activated, phosphorylated Chk1 kinase (Chk1‐P); (F): medulloblastoma with variable nuclear staining for activated, phosphorylated ATR kinase (ATR‐P). The scale bars in A–F represent 50 µm. Yellow arrows in (A) and (D) mark Purkinje cells.

Given the robust occurrence of the γH2AX marker in our cohort, we next focused on the two major apical DNA damage signalling kinase modules, namely ATM (and its target kinase Chk2) that responds to DNA double‐strand breaks and ATR (and its target kinase Chk1) that becomes activated by replication stress and a variety of DNA structures and lesions that occur mainly in S phase (Bartek *et al*., 2012; Jackson and Bartek, 2009; Lecona and Fernandez‐Capetillo, 2014; Lukas *et al*., 2011). To assess whether one or possibly both of these DDR signalling cascades are activated in the medulloblastoma biopsy specimens, we employed well‐characterized and validated antibodies to activated (phosphorylated) forms of ATM (ATM‐P), Chk2 (Chk2‐P), ATR (ATR‐P) and Chk1 (Chk1‐P) using the same method as for the detection of γH2AX. Whereas none of these antibodies showed any positive staining on the control sections of human normal cerebellum, they all showed nuclear staining patterns of variable intensity and extent among the medulloblastomas. Representative images are shown in Fig. 2, and the overall results are summarized as part of Table 2. Notably, while both the ATM‐Chk2 and ATR‐Chk1 signalling cascades were activated to some extent in each and every case, there were five tumours that showed a higher level of ATR‐Chk1 axis activation compared to ATM‐Chk2 (cases no. 2, 3, 10, 15, 17 in Table 2), and the remaining cases displayed a comparable degree of ‘activity’ for both kinase modules (Table 2). Parallel control immunohistochemical analyses confirmed the presence of ATM and Chk2 proteins, and also nuclear positivity for the key DNA damage mediator protein 53BP1 in at least 70% cells of all medulloblastomas as well as in control normal brain tissues (Table 2 and data not shown), suggesting that defects of these tumour suppressor proteins, at least at the level of protein expression and nuclear localization, are likely rare. On the other hand, a subset of medulloblastoma cells in all tumours displayed nuclei with larger foci, or ‘nuclear bodies’ of 53BP1 (see inset in Fig. 2, panel C). These larger structures are reminiscent of the G1‐phase bodies that indicate the history of replication stress experienced by the cell in the previous cell cycle, a scenario that may lead to mitotically born chromosomal breaks, which are then recognized and surrounded by the 53BP1 bodies in G1 daughter cells (Lukas *et al*., 2011).

We conclude from these analyses that both the ATM‐Chk2 and ATR‐Chk1 kinase modules are widely ‘spontaneously’ activated among human paediatric medulloblastomas, thereby suggesting that endogenous DNA double‐strand breaks and replication stress and/or replication‐associated DNA lesions occur commonly in these malignancies, even prior to any radiochemotherapy.

### 3.3 The p53 tumour suppressor, proliferation index and oxidative DNA damage

As the DNA damage checkpoints converge on stabilization and activation of the p53 tumour suppressor protein, we next examined the p53 staining patterns in our cohort of medulloblastomas. Furthermore, to complement the assessment of biological parameters highly relevant to replication stress and oxidative DNA damage, we also examined the proliferation index (as a percentage of tumour cells positive for the Ki67 proliferation marker) and the presence and subcellular localization of the major oxidative DNA lesions, detected by an antibody to 8‐oxoguanine (8‐oxoG), both well‐established biomarkers used also in our previous studies (Bartkova *et al*., 2010; Kurfurstova *et al*., 2016).

**Figure 3:**
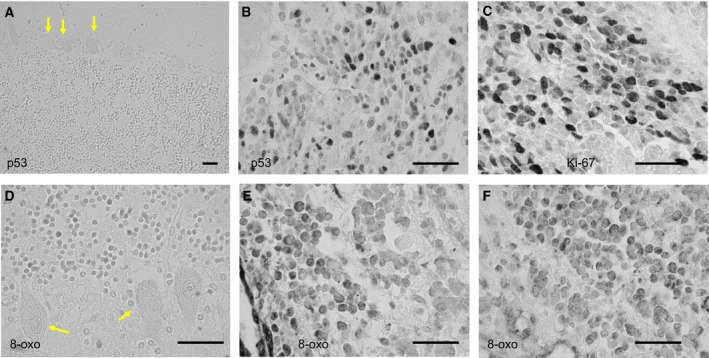
Immunohistochemical detection of p53, Ki67 proliferation marker and oxidative DNA lesions (8‐oxoguanine), using the staining Protocol ‘A’. Normal cerebellum shows undetectable p53 staining (A), while medulloblastomas display a range of heterogeneous positivity for p53, here one of the most positive cases (B); high proliferation rate in a medulloblastoma, indicated by staining for Ki67 (C); the oxidative DNA lesions were barely detectable in normal cerebellum when stained for 8‐oxoG (D), in contrast to a wide range of oxidative damage (8‐oxoG) seen in medulloblastomas (E, F). The scale bars in A–F represent 50 µm. Yellow arrows in (A) and (D) mark Purkinje cells.

The percentage of tumour cells with elevated nuclear p53 protein varied between 0.5% and 40% in our cohort (Table 2), in contrast to normal cerebellum in which p53 was undetectable (Fig. 3A,B). The fact that fractions of medulloblastoma cells showed elevated p53 is in agreement with the active ongoing DNA damage signalling, as p53 is a substrate for all four DDR kinases examined, and the resulting phosphorylations contribute to stabilization and activation of p53. Based on the commonly employed cut‐off value of 20% of p53‐positive cells (Fagerholm *et al*., 2008), it is very likely that at least the three medulloblastomas (no. 10, 22 and 24, Table 2) with the highest percentage of positive nuclei harbour mutant p53. All these three p53‐aberrant tumours showed very high degrees of DNA damage checkpoint activation (γH2AX marker and ATR/ATM pathways activation), consistent with the notion that DNA damage signalling activates p53 and one of the pathogenic routes to escape from such checkpoint pressure in order to progress towards full malignancy is selection of tumour cells with mutant p53 (Halazonetis *et al*., 2008). On the other hand, considering the overall high extent of ATM‐Chk2 and ATR‐Chk1 activation, we were surprised to find many tumours, particularly of the molecular subtype 3/4 (non‐Wnt2, non‐Shh), in which detectable p53 protein was limited to a very minor proportion (0.5–5%) of cancer cells (see Table 2). The likely explanation(s) for these apparently counterintuitive results, namely ‘discrepancy’ between the pronounced DNA damage signalling on the one hand and the often very modest p53 elevation on the other, may reflect the unorthodox mechanisms of p53 inactivation other than p53 mutations in medulloblastomas, as explained in more depth in the Section 4.

The Ki67‐based proliferation index was widely variable among the tumours in our cohort, from the lowest 14% to the highest 60% value (Table 2, and Fig. 3C), overall consistent with the aggressive and rapidly proliferating nature of medulloblastomas. This parameter also correlated well with the extent of DNA damage signalling (γH2AX marker) and pronounced activation of the ATR‐Chk1 axis that responds to replication stress. Indeed, all the six cases with the highest proliferation index (between 40 and 60%) showed the highest degree of DNA damage activation (category ‘D’, see Table 2).

Oxidative stress can also contribute to endogenous DNA damage and we have previously reported aberrantly enhanced oxidative damage (8‐oxoG levels) in human glioblastomas compared to lower‐grade gliomas (Bartkova *et al*., 2010). In our present analysis, the 8‐oxoG marker was barely detectable in normal human cerebellum (Fig. 3D) yet it was aberrantly enhanced and varied greatly, between 1% and 60% of strongly positive cancer cells in the individual tumours (Table 2). Notably, the immunohistochemical staining signal for 8‐oxoG was commonly localized to cytosol, yet also some cancer cell nuclei were positive (Fig. 3E,F), an observation consistent with both DNA and RNA being subject to oxidative lesions, and both nuclear and mitochondrial genomes being potentially targeted. In cases with pronounced oxidative damage, we observed particularly strong 8‐oxoG staining in nuclei of endothelial cells lining some tumour blood vessels (Fig. 3E, left bottom area of the image), an intriguing phenomenon that deserves further investigation.

These results indicate a wide variability in terms of proliferation rates, extent of p53 stabilization and oxidative damage among human medulloblastomas, features that are further considered in the Section 4, along with the other parameters examined in this study.

### 3.4 Immunohistochemical detection of HCMV proteins in medulloblastomas

**Figure 4:**
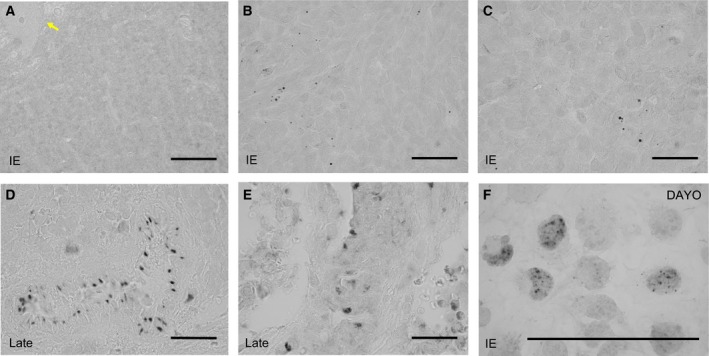
Immunohistochemical detection of HCMV proteins using the staining Protocol ‘B’. HCMV immediate early protein is undetectable in normal human cerebellum (A), yet positive in subsets of tumour cells in two medulloblastomas (B, C); patterns of HCMV late protein in two medulloblastomas, with the staining signal localized to blood vessel endothelium (D, E); example of HCMV immediate early protein detection in nuclei of cultured human medulloblastoma cell line DAOY (F). The scale bars in A–F represent 50 µm. Yellow arrow in (A) marks a Purkinje cell.

**Figure 5:**
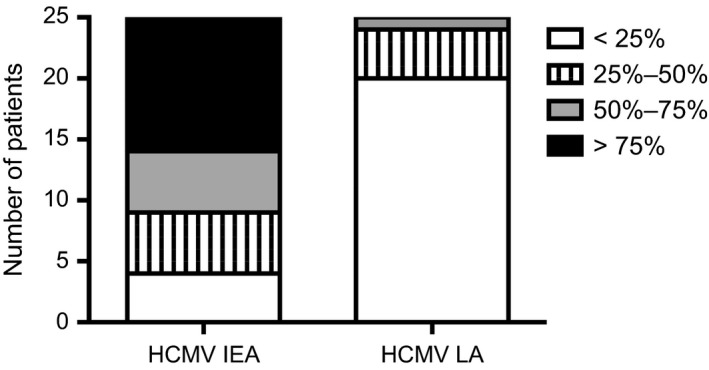
Graphical summary of immunohistochemical detection of HCMV immediate early and late protein, respectively, in the same cohort of medulloblastoma tumours (*n* = 25), yet using the Protocol ‘C’. See Section 2 and main text for details.

In order to reassess the detection of HCMV immediate early and late proteins, we compared three immunohistochemical protocols that differed in the antigen retrieval buffers and also other steps, such as including or not nuclear counterstaining, or reaction enhancement, as specified in the Section 2. Overall, we were able to detect both the HCMV immediate early protein IE72 and the HCMV late protein in variable proportions of medulloblastoma tumour cells, in contrast to the lack of any staining in normal cerebellum. The fraction of cells per lesion that showed positive staining when Protocol B was used was variable and ranged between 1 and 10% in all cases. Examples of representative images obtained with the Protocol B (using the Tris buffer pH 9.0 for unmasking, combined with the sensitive immunoperoxidase method and the lack of nuclear counterstaining), are shown in Fig. 4A–E. While the focal staining for the HCMV immediate early IE72 protein was very heterogeneous and detectable in both nuclei and occasionally cytosol of a fraction of medulloblastoma cells (Fig. 4B,C), the late protein was particularly prominent in blood vessel endothelium in some areas of the tumours, mostly in the form of larger cytosolic structures (Fig. 4D,E). On the other hand, using the standard Protocol ‘A’ that includes the citrate buffer (pH 6.0) for antigen unmasking and which is a standard method reliable for the detection of the DDR markers presented in Table 2, proved suboptimal for staining of the HCMV proteins (data not shown). The successful Protocol ‘B’ was also applied to immunoperoxidase staining of cultured and fixed medulloblastoma cell lines, and revealed a minority fraction of positive cells for HCMV immediate early protein in the form of nuclear foci (Fig. 4F), reminiscent of the pattern seen in subsets of medulloblastoma cells on sections from the clinical specimens (Fig. 4B,C). Analogous staining of medulloblastoma cell lines for the HCMV late protein was negative, arguably consistent with the positivity on the clinical specimens being largely confined to endothelium. The third immunohistochemical method (referred to as Protocol ‘C’ in this study) that was used here was the established protocol based on the original Cobbs’ method and previously used successfully to detect HCMV proteins in diverse types of human tumours including an independent cohort of human medulloblastomas (Baryawno *et al*., 2011). The results obtained with this latter method are expressed here in a graphical form, based on the previously validated semiquantitative scale (Fig. 5).

What we conclude from this comparative analysis is that the successful detection of HCMV proteins by immunohistochemistry on archival paraffin sections does depend on the tissue processing and staining protocol used, an important notion that is further considered in the Section 4. As both the HCMV protein detectability and the extent of activated DNA damage signalling were virtually universal among our cohort, and the overall number of cases in any subgroups was limited, we did not find any clear correlation between the proportion of cancer cells positive for DDR activation versus HCMV protein expression. On the other hand, clinical specimens provide just a static one‐time window into the time‐dependent complex process of tumour development, and therefore, we wished to examine also human cultured cells, as a model system more amenable to experimentation, and presented in the following subsection of the Section 3.

### 3.5 Medulloblastoma cell lines: replication stress and impact of HCMV infection

**Figure 6:**
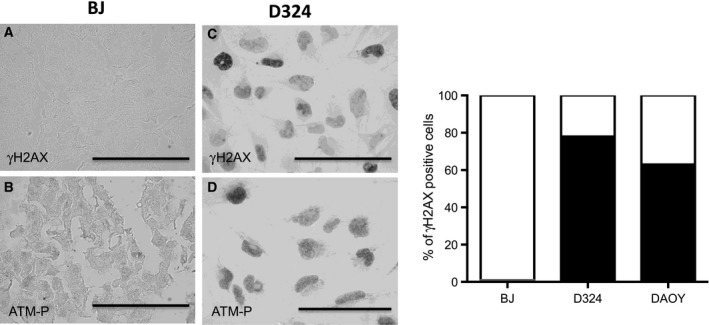
Immunoperoxidase detection of DNA damage‐response markers in cultured human control diploid fibroblasts (strain BJ) and medulloblastoma cells (D324 cell line). (A): Negative staining for γH2AX in BJ fibroblasts; (B): negative staining for activated/phosphorylated ATM kinase in BJ fibroblasts; (C): D324 medulloblastoma cells with nuclei variably positive for γH2AX; (D): D324 medulloblastoma cells with nuclei variably positive for activated/phosphorylated ATM kinase. (E): Graphical summary for percentage of cells positive for the DDR marker γH2AX in the indicated cell types (negative BJ fibroblasts, heterogeneously positive medulloblastoma cell lines D324 and DAOY); graphs represent results from one of three reproducible experiments.

To address the potential involvement of endogenous DNA damage checkpoint activation and replication stress in human medulloblastomas, as indicated by the robust γH2AX marker and strong activation of the ATM‐Chk2 and ATR‐Chk1 signalling cascades on the clinical specimens (see Section 3.2 above), along with any relationship to HCMV, we turned to an established model of the human medulloblastoma cell lines D324 and DAOY, in some experiments complemented by direct infection with HCMV. First, we could show that analogous to the clinical specimens, a large proportion of both D324 and DAOY cells feature nuclear positivity for the general DDR activation marker γH2AX and activated ATM, Chk2, Chk1 and ATR kinases when cultured under unperturbed growth conditions (Fig. 6 C, D, E and data not shown), hence reflecting endogenous DNA damage and likely replication stress. None of these markers were detectable in parallel cultures of control normal human diploid BJ fibroblasts (Fig. 6A,B,E).

**Figure 7:**
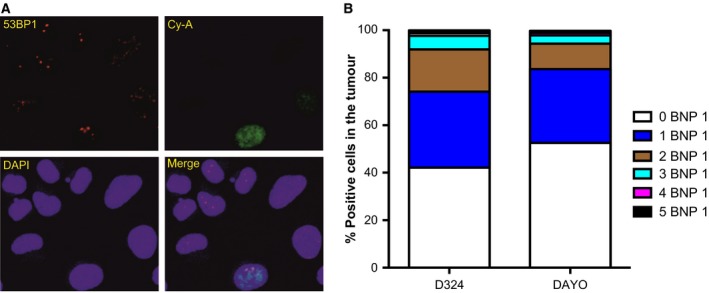
Confocal immunofluorescence microscopy analysis of the G1‐phase nuclear bodies formed by the DDR protein 53BP1 in human medulloblastoma cell lines exponentially grown under unperturbed conditions. (A): Example of immunofluorescence double staining in D324 cells, for 53BP1 (red) and cyclin A (green), with DAPI DNA counterstaining (blue). For counts of G1‐phase 53BP1 bodies indicating replication stress, the cyclin A‐positive (S‐G2 phase) cells were excluded. (B): Graphical summary of the categories of medulloblastoma G1‐phase cell nuclei showing 0–5 53BP1 bodies per nucleus, in the two human medulloblastoma cell lines as indicated.

To further examine the possibility of endogenous replication stress suggested by the elevated and persistent activation of the ATR‐Chk1 kinase axis, we next assessed the occurrence and frequency of the so‐called 53BP1 bodies in G1‐phase cells (Lukas *et al*., 2011) in unperturbed exponentially growing cell populations. This convenient and commonly used biomarker indicates ongoing endogenously evoked replication stress experienced by the examined cells during their S‐G2‐M traverse in the preceding cell cycle, and manifested by mitotically formed DNA breaks due to genomic loci such as fragile sites that remained unreplicated when the cells reached the mitotic division and hence became fractured during mitosis. Such DNA breaks are then recognized and kept insulated by the DNA damage‐response ‘factories’ in the newly born G1 cells, and until the next S phase, and conveniently detected as the ‘bodies’ or larger foci positive for the 53BP1 DDR protein in cells negative for cyclin A expression, hence residing in the G1 phase of the cell cycle (Burrell *et al*., 2013; Lukas *et al*., 2011). Indeed, using double immunofluorescence staining for 53BP1 and cyclin A (marker of S/G2 cells), we could detect variable numbers of such 53BP1 nuclear bodies in the G1 cells among the exponentially growing D324 and DAOY medulloblastoma cell lines (Fig. 7). The frequency of this replication stress indicator was clearly above the rather rare occurrence seen in normal diploid cells (Lukas *et al*., 2011) (and our unpublished data).

**Figure 8:**
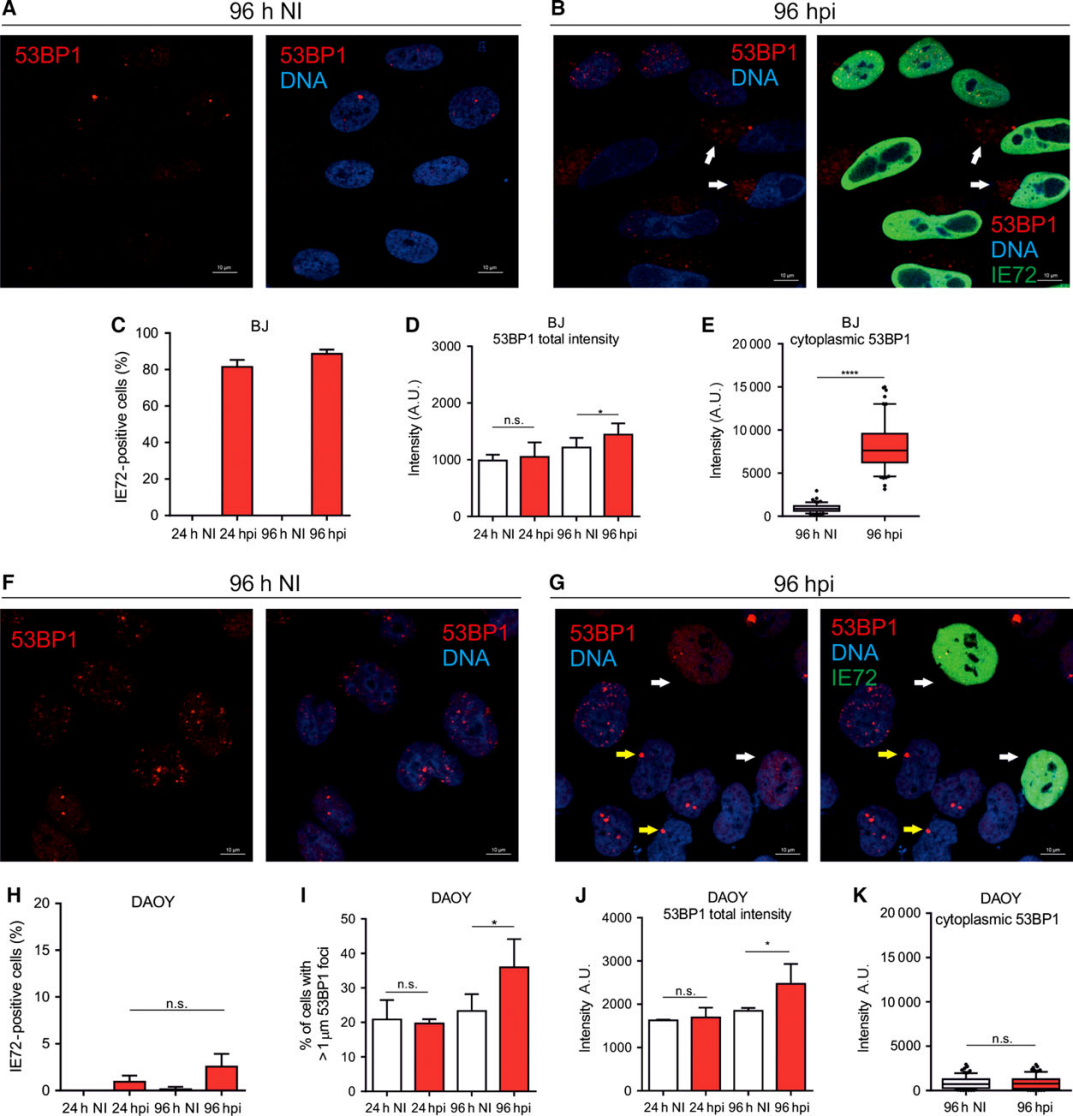
Human cytomegalovirus infection induces 53BP1 expression but not cytoplasmic mislocalization in medulloblastoma cells; (A–E) BJ cells were infected with VR1814 HCMV (MOI 3) and analysed for the expression of 53BP1 at different time postinfection (pi). (A,B) Representative photomicrographs of 53BP1 (red) and IE72 (green) immunostaining in noninfected (NI) cells (A) and at 96hpi (B) are shown. White arrows indicate cytoplasmic 53BP1 expression. (C) The graph represents the percentage of IE72(+) cells together with the standard deviation (STDV) from the mean at 24 h (24h NI *n* = 429; 24hpi *n* = 678) and 96 h (96 h NI *n* = 488; 96hpi *n* = 709). (D) The graph represents 53BP1 total intensity together with STDV at 24 h (24 h NI *n* = 318; 24hpi *n* = 338) and 96 h (96 h NI *n* = 328; 96 hpi *n* = 325). (E) The graph represents 53BP1 cytoplasmic intensity together with STDV at 96 h (96h NI *n* = 123; 96hpi *n* = 122). (F–K) DAOY cells were infected with VR1814 HCMV (MOI 3) and analysed for the expression of 53BP1 at different time postinfection. (F,G) Representative photomicrographs of 53BP1 (red) and IE72 (green) immunostaining in noninfected (NI) cells (F) and at 96hpi (G) are shown. White arrows indicate the lack of cytoplasmic 53BP1 expression. Yellow arrows indicate > 1 μm 53BP1 foci. (H) The graph represents the percentage of IE72(+) cells together with STDV at 24 h (24 h NI *n* = 520; 24hpi *n* = 852) and 96 h (96 h NI *n* = 189; 96hpi *n* = 1174). (Note the scale on the *y*‐axis). (I) The graph represents the percentage of cells with 53BP1 foci >1 μm together with STDV at 24 h (24 h NI *n* = 773; 24hpi *n* = 832) and 96 h (96 h NI *n* = 745; 96hpi *n* = 1028). (J) The graph represents 53BP1 total intensity together with STDV at 24 h (24 h NI *n* = 773; 24hpi *n* = 832) and 96 h (96 h NI *n* = 745; 96 hpi *n* = 1028). (K) The graph represents 53BP1 cytoplasmic intensity together with STDV at 96 h (96h NI *n* = 148; 96hpi *n* = 155). Scale bars, 10 μm. *P < 0.05, *^***^ *P* < 0.0001, n.s. – statistically not significant, Student's *t*‐test.

Together with the immunohistochemical results on clinical specimens of our medulloblastoma cohort, the findings of enhanced 53BP1 bodies and hence endogenous replication stress in the medulloblastoma cell lines raised the question whether HCMV infection might in any way impact the extent of replication stress and/or the formation of the 53BP1 bodies. To address this issue, we directly infected the control human BJ diploid fibroblasts and the DAOY medulloblastoma cell line with the commonly used HCMV clinical isolate‐like strain VR1814, and examined the expression of the viral immediate early protein IE72 and the endogenous cellular DDR factor 53BP1, at times 0h, 24 h and 96 h postinfection. As can be seen from the representative examples of double immunofluorescence images in Fig. 8A,B,C, the IE72 protein became clearly detectable in some 90% of the infected BJ fibroblasts, consistent with these cells being easily infectable and permissive for viral replication, and the prototype cell for the production of infectious virus. Quantitative fluorescence image analysis of the total cellular 53BP1 showed a moderate induction of 53BP1 level by 24 h postinfection, and this trend became significant by 96 h postinfection (Fig. 8D). Notably, the nuclear expression of the viral IE72 protein was accompanied by a massive mislocalization of the endogenous 53BP1 protein from its physiological nuclear pattern into cytosol (Fig. 8B). This shift in HCMV‐infected as compared to mock‐infected control cells was robustly apparent upon both microscopic inspection and quantification, as the difference at 96 h postinfection was highly statistically significant (*P* < 0.0001, see Fig. 8E). Contrary to the BJ cells, the infection of the DAOY medulloblastoma cells with the same virus strain and titre was much less efficient (Fig. 8H), yet sufficient to obtain enough strongly IE72‐expressing cells for the analyses of the endogenous 53BP1 protein. Notably, analysis of 53BP1 revealed two striking and unexpected phenomena. First, while the overall cellular intensity of 53BP1 staining increased in a similar manner as in the infected BJ cells (compare Fig. 8J and 8 D), the physiological nuclear localization of 53BP1 remained unaltered in the DAOY cells regardless of the HCMV infection, even in the nuclei with strong IE72 expression by 96h postinfection. In other words, there was no sign of HCMV‐induced cytosolic relocalization of 53BP1 (Fig. 8F,G,K), in sharp contrast to the BJ cells (Fig. 8B,E). Second, quantitative image analysis including measurements of the size of the 53BP1 foci/bodies showed a significantly increased proportion of DAOY cells containing the nuclear 53BP1 bodies with the diameter over 1 um, a characteristic feature of the replication stress‐associated 53BP1 bodies in G1 cells (Fig. 8I). What was equally intriguing was the fact that the latter enhancement of the cellular subset with the 53BP1 bodies was prominent among the uninfected DAOY cells within the same cell population, while the minority of HCMV‐positive nuclei (marked by detectable IE72) appeared to lack 53BP1 bodies. Thus, there was a distinctly different localization pattern of 53BP1 in HCMV‐infected cells versus the uninfected cells in the virus‐infected DAOY cell cultures, which implies that the formation of 53BP1 bodies may be affected by soluble factors produced by virus‐infected cells.

Considering the results of cell culture experiments in the context of the immunohistochemistry on clinical specimens, we conclude that human medulloblastomas show signs of endogenous replication stress including chronic ATR‐Chk1 activation and formation of the 53BP1 bodies. Furthermore, we observed an unorthodox response of medulloblastoma cells to experimental HCMV infection, including enhanced frequency of the replication stress‐characteristic 53BP1 nuclear bodies in a cell‐non‐autonomous manner. The potential pathophysiological significance of these unexpected findings is discussed below in the context of role(s) HCMV could play in tumorigenesis.

## 4 Discussion

The findings from this study provide multiple insights into the status of the DNA damage‐response machinery and its emerging links with the HCMV infection as factors that likely impact the genetic landscape and biology of human medulloblastomas during the process of tumorigenesis. Besides complementing the previous study on HCMV in human medulloblastomas (Baryawno *et al*., 2011), we wish to highlight the conceptual advances and potential pathophysiological relevance of three related findings from the data set that we report here: (a) persistent and therapy‐unrelated DNA damage‐response activation as a candidate biological barrier in paediatric medulloblastomas, including the unorthodox involvement of the p53 tumour suppressor; (b) prevalence and technical aspects of HCMV detection in archival clinical samples; and (c) our evidence for replication stress as a candidate driver of endogenous DNA damage in medulloblastomas, and a potential cross‐talk of HCMV with the host cell DDR machinery in response to replication stress.

The first major finding is that the genome integrity control network including DNA damage checkpoint signalling is constitutively and ‘spontaneously’ activated in basically all medulloblastoma cases in our cohort, at stages preceding any radiochemotherapy. The evidence for this conclusion is provided by the immunohistochemical patterns of the DNA damage signalling marker γH2AX, as well as activated forms of the ATM, ATR, Chk1 and Chk2 kinases on the sections of all medulloblastomas in our cohort, contrary to the absence of these DDR markers on sections of normal cerebellum. This robust overall result strongly suggests that the concept of the DNA damage‐response activation as an intrinsic biological barrier against activated oncogenes and tumour progression (Bartek *et al*., 2007; Bartkova *et al*., 2005b; Gorgoulis *et al*., 2005; Halazonetis *et al*., 2008) is applicable also to medulloblastomas. One reason this issue is particularly pertinent to be elucidated for medulloblastomas was the sharp difference among the brain tumours examined so far, in that gliomas (Bartkova *et al*., 2010), but not paediatric intracranial germ‐cell tumours (Bartkova *et al*., 2014), show robust DDR activation in clinical tumour specimens. Overall, the emerging scenario that considers also our present data on medulloblastomas is that the critical factors that dictate whether or not, and to what extent, is the DDR checkpoint barrier activated reflect the cell origin of the particular brain tumour and/or the oncogenes that deregulate the replication machinery leading to enhanced replication stress and DNA damage. While we are unable to point to any specific oncogene that triggers the DDR checkpoint activation in human medulloblastomas, we did address this issue in part, by interrogating the molecular subtypes of the tumours in our cohort. The subtyping has been carried out according to the current international schemes that reflect, at least in part, also the major oncogenic events believed to drive the four major molecular subtypes (Gajjar and Robinson, 2014; Taylor *et al*., 2012). Although the molecular subtyping could be performed for only 15 of the 25 medulloblastomas in our cohort, due to the limitation of the available tissue, and our analyses could not distinguish between the molecular subtypes 3 and 4 (Table 2), we observed the DNA damage‐response pathway activation in lesions of subtypes 1, 2, as well as the largest group of subtypes 3/4. Therefore, it is likely that multiple oncogenic events, and also loss of some tumour suppressors, may contribute to cause endogenous DNA damage, activate the DDR checkpoints and eventually also fuel genomic instability, a prominent feature of both glioblastomas and medulloblastomas (Bartkova *et al*., 2010; Godek *et al*., 2016; Northcott *et al*., 2012). Of note, amplifications and overexpression of either Myc or MycN oncogenes are relatively common among all subtypes of medulloblastoma, and Myc has been already implicated as a trigger of cellular replication stress (Gajjar and Robinson, 2014; Maya‐Mendoza *et al*., 2015; Northcott *et al*., 2012; Pui *et al*., 2011). On the other hand, further experiments are needed to elucidate the potential mechanisms through which other oncogenes implicated in medulloblastoma pathogenesis, such as those in the Wnt and Shh pathways or aberrant epigenetic chromatin modulators (Gajjar and Robinson, 2014; Northcott *et al*., 2012; Pui *et al*., 2011), might cause replication stress.

A related and very intriguing notion is the apparent discrepancy between our present finding of the widespread activation of the DNA damage checkpoint, yet at the same time in many cases accompanied by only very modest if any stabilization of the key downstream target of these checkpoints, the p53 tumour suppressor (Table 2). We only found three of the 25 cases in our cohort featuring large fractions of medulloblastoma cells with elevated p53, the pattern that almost invariably corresponds to point mutations of the p53 gene (Fagerholm *et al*., 2008; Iggo *et al*., 1990). Indeed, mutations of the *p53* gene are much less common among medulloblastomas (Gajjar and Robinson, 2014; Northcott *et al*., 2012; Pui *et al*., 2011) compared to major epithelial cancers or gliomas (Halazonetis *et al*., 2008; Ranjit *et al*., 2015). This combination of robust DDR checkpoint activation yet rare p53 mutations seems even more counterintuitive given that the activated DDR checkpoints create a pressure for selecting cancer cell clones with mutant p53 as a way to escape the oncogene‐induced, p53‐mediated senescence or cell death of the nascent tumour cells that otherwise often results from the chronic activation of the ATM‐Chk2/ATR‐Chk1‐p53 pathway (Halazonetis *et al*., 2008). We propose that the possible solution to this puzzle might lie in the unorthodox ways in which p53 is inactivated in medulloblastomas. The two major mechanisms in which this occurs, apart from the selection for p53 mutations, are i) deletion of chromosome 17p on which the *p53* gene resides, often in a process that leads to the occurrence of isochromosome 17 in human medulloblastomas (De Smaele *et al*., 2004), and ii) frequent overexpression of the phosphatase Wip1 that efficiently dephosphorylates and hence deactivates wild‐type p53 (Castellino *et al*., 2008). Collectively, these mechanisms and a relatively few cases of genuine p53 mutations may account for the otherwise rather unorthodox dichotomy between strong DDR checkpoint signalling that is not accompanied by more robust p53 elevation, a pattern we identified in our immunohistochemical analysis of clinical medulloblastoma specimens.

The second major goal of this work was to address the current debate in the field of HCMV prevalence and HCMV's potential tumour‐modulatory role, which is referred to in the literature as oncomodulation (Michaelis *et al*., 2009, 2012). While the bulk of studies agree on detectability of HCMV immediate early and some other HCMV proteins in most if not all types of human cancer (Cobbs, 2013, 2014; Soderberg‐Naucler and Johnsen, 2012), there have also been reports, some of which are recent and focused on brain malignancies, of failed attempts to reliably detect the HCMV proteins by immunohistochemistry (Baumgarten *et al*., 2014; Sardi *et al*., 2015; Yamashita *et al*., 2014). We believe the value of our present results as a contribution to this field is the parallel comparison of three immunohistochemical protocols, as a helpful guide to select the appropriate method. Our data revealed that using two of the three approaches used, called Protocol ‘B’ and Protocol ‘C’, respectively, we were able to detect the HCMV immediate early as well as late protein in virtually all cases, albeit the fraction of positive cells was small in some tumours (see Section 3 and Figs 4 and 5). One of the very critical aspects of the detection appears to be the antigen unmasking step, which relied on heat treatment of sections in either citrate buffer pH6 (Protocol A), Tris buffer pH9 (Protocol B) or enzymatic processing (Protocol C) of the tissue sections. As the Protocol C was used before and is very similar to the widely used method pioneered by the Cobbs laboratory (Rahbar *et al*., 2012, 2013), here we highlight only some features of our Protocol B, which was employed for HCMV detection for the first time in our present analysis. We believe the replacement of the citrate buffer for the Tris buffer of pH9 during the antigen unmasking step is very important, and this buffer was chosen based on the recent successful detection of HCMV proteins by Bianchi and colleagues (Bianchi *et al*., 2015), who, however, used a different staining protocol after antigen unmasking. Furthermore, our Protocol B involves the sensitive staining method including the reaction enhancement step, and unlike other methods, it avoids nuclear counterstaining of the sections. The latter feature is useful to spot even relatively delicate staining signals in the nuclei, useful for foci detection of the DDR proteins but also for the HCMV immediate early protein, for example. On the other hand, evaluation of immunohistochemistry results obtained with Protocol B requires an experienced pathologist, as the lack of counterstaining makes the recognition of structures and cells more challenging (see Fig. 4 as examples). With regard to the HCMV staining patterns we obtained, the apparent prevalence of the immediate early protein in cancer cells (both nuclear and cytosolic) compared to the largely endothelial and cytosolic staining for the HCMV late protein suggests that HCMV is most likely not replicating in tumour cells, but in endothelial cells in the tumour. *In vitro*, it is well known that HCMV does not replicate and produce infectious virus in most tumour cell lines, but rather produce nonpermissive infections with expression of IE proteins and limited viral genes. Genetic analyses of HCMV in glioblastoma further support that HCMV is not replicating in this tumour. Instead, and supported by data provided herein, we envision that HCMV affects the tumour by limited number of viral proteins with strong oncomodulatory functions that will affect the tumour phenotype. These may include epigenetic regulation, cell cycle control, induced stemness, a Warburg effect‐like reprogramming of cellular metabolism, chromosomal instability and mutations, induced angiogenesis and immune evasion strategies in virus‐infected tumour cells.

The third issue we wish to highlight is the first evidence we present here for chronic replication stress in medulloblastomas, and especially the intriguing and surprisingly clearly differential modes in which HCMV impacts the replication stress‐responding mechanism of the host human cells, when normal diploid cells are compared with medulloblastoma cells in functional experiments. The main results indicating that human medulloblastomas suffer from endogenously triggered and chronic replication stress are as follows: (a) robust and ongoing activation of the ATM‐Chk2 and especially of the ATR‐Chk1 kinase signalling cascades in the clinical specimens of human medulloblastomas; (b) analogous chronic DDR checkpoint signalling by ATM/ATR detected in the human medulloblastoma cell lines under unperturbed growth conditions; and (c) prominent formation of the replication stress‐responding 53BP1 nuclear bodies observed in subsets of medulloblastoma cells in both clinical specimens and the cultured medulloblastoma lines. The potential endogenous triggers of such sustained signalling in terms of candidate oncogenic events behind this phenomenon have been mentioned earlier in the Section 4. In addition, what is very intriguing is the functional impact of the experimental HCMV infection in human cells on the fate of 53BP1, the key DNA damage‐response adaptor protein and fundamental component of the replication stress‐responding 53BP1 bodies that we saw in both the tissue sections and cell culture models. The striking and fundamental difference upon infection of cells by HCMV in our experiments was that 53BP1 was almost completely mislocalized from the nucleus to the cytosol in the IE72‐expressing, HCMV‐infected BJ normal cells, in sharp contrast to no such relocation, and diffuse 53BP1 expression in IE72‐positive HCMV‐infected medulloblastoma cells, within the HCMV‐infected cultures (Fig. 8). While we can presently only speculate about why does the HCMV infection evoke such dramatically different fates of 53BP1 in normal fibroblasts versus medulloblastoma cells, at least three scenarios come to mind. First, the BJ diploid cells are permissive for HCMV replication and active removal of the DDR factor 53BP1 from nuclei during the viral replication might allow the viral genomes to be rapidly replicated without the host cell DNA damage‐response machinery interfering with this process. The second reason would be simply some kind of passive blockade of, for example, nuclear cytoplasmic transport in the virus‐replicating cells, yet in this case such mechanism would not operate for some reason in medulloblastoma cells. Third, it is possible that the HCMV presence and its interplay with the host cell genome and DNA damage checkpoint and/or repair pathways cause enhanced chromosomal instability in cancer cells such as medulloblastomas in our experiments, and therefore, it might be beneficial for the chronic viral presence in tumour cells to preserve the function of 53BP1 in promoting repair of DNA double‐strand breaks that do occur with enhanced frequencies in cancer cells (Halazonetis *et al*., 2008). In addition, we show that HCMV infection in fact leads to enhanced levels of 53BP1 (Fig. 8). Given that 53BP1 shifts the balance of the repair of DNA double‐strand breaks towards the more error‐prone pathway of nonhomologous end‐joining, at the expense of the more accurate homologous recombination (Lord and Ashworth, 2012) (Jackson and Bartek, 2009), such altered balance among the key cellular pathways of DNA break repair would then likely fuel the chromosomal instability of the cancer cells over time, consistent with the high degree of genomic instability known to occur in medulloblastomas, among other malignancies. Yet another unexpected aspect of our functional cell culture experiments was the observed increase in the 53BP1 bodies in the infected cell populations of medulloblastoma cells, and there was the evidence that this effect was apparent among the cells that did not show expression of the viral IE72 protein. This suggests that some cell‐non‐autonomous mechanism, possibly a viral factor or HCMV‐triggered host cell factor, might be, for example, secreted from the minor population of the HCMV‐infected cancer cells and impact the surrounding noninfected cells to also trigger the enhanced 53BP1 abundance and formation of 53BP1 bodies. Such possibility would indicate a trend for enhanced replication stress experienced by the overall tumour population, even if only a fraction of the cells was directly expressing the viral proteins. This last scenario, while in need of thorough experimental testing in future, would be tempting in that also in the clinical specimens HCMV was always present only in a fraction of the medulloblastoma cells. Regardless of the precise mechanisms involved, our present results on the emerging interplay between HCMV and the replication stress‐response machinery of the cancer cells raise a host of important questions relevant to genomic integrity and tumorigenesis, issues that will need to be addressed by dedicated experiments in future. It is also intriguing that HCMV causes the increase in the larger‐in‐size 53BP1 bodies (Fig. 8), somewhat reminiscent of such larger 53BP1 bodies that we previously detected in human tumours linked to human papillomavirus infection (Gudjonsson *et al*., 2012).

Last but not least, it is plausible that our present results turn out to be relevant also for the therapeutic responses of patients with medulloblastoma to radiation or chemotherapy. We would argue that under conditions of persistent and robust signalling of the ATM‐Chk2 and ATR‐Chk1 DNA damage checkpoints that we report here, the tumour cells must remarkably adapt in order to proliferate and avoid cell cycle arrest (senescence) or cell death otherwise evoked by such checkpoint signalling (Bartek *et al*., 2007, 2012; Halazonetis *et al*., 2008). Notably, we see the chronic DDR signalling and yet concomitantly relatively high proliferation index (monitored by the Ki67 marker, Table 2) in the medulloblastoma biopsies taken before any genotoxic radiochemotherapy was initiated. Therefore, one could presume that such adapted tumour cells would also be more resistant to other insults that trigger the DNA damage‐induced ATM/ATR‐mediated responses, and therefore, such tumour population might already contain clones of cells a priori resistant, or more easily further adaptable, to the later clinical administration of genotoxic insults in the form of radiation or chemotherapy. In other words, the phenomenon of endogenously high DDR checkpoint activation that we report here for paediatric medulloblastomas, along with the impact of HCMV on the replication stress response of the tumour cells, may be intimately linked to the well‐known adverse clinical problem of resistance to standard‐of‐care nonsurgical treatment modalities routinely used in neurooncology today.

## Author contributions

JB Jr., JiB, JB and CSN conceived and designed the project. JB Jr., OF, JMMM, AMM, HB and AS acquired the data. JB Jr., OF, JMMM, AMM, AR, HB, MS, GS, AS, JiB, JB and CSN analysed and interpreted the data. JB Jr., JiB, JB and CSN wrote the manuscript.
